# Supplementary material for: SAQ training on sprint, change-of-direction speed, and agility in U-20 female football players
Source: PLoS One. 2024 Mar 13;19(3):e0299204. doi: 10.1371/journal.pone.0299204 (PMC10936847; doi:10.1371/journal.pone.0299204)
Supplement: Attachment — Submitted filename: Plos_SAQ_responses_to_reviewers_R1.docx [file pone.0299204.s002.docx]

PONE-D-22-09664 “SAQ training on sprint, change-of-direction speed, and agility in U-20 female football players”

Point-by-point responses to the reviewers’ comments

Overall comments: We would like to thank the editors for allowing us to submit the revised draft of the manuscript and the reviewers for providing us with constructive and insightful comments. Your suggestions were useful to improve the current manuscript. We studied the feedback carefully to address the reviewers’ concerns and worked on to strengthen those areas to reflect most of the comments by the reviewers. Please see the point-by-point descriptions of every single change we made in the right column below (next to the actioned one), and we marked the edits in the manuscript using the track changes function in Word.

| Reviewers’ comments | Responses | Actioned |
| --- | --- | --- |
| Journal Requirements:  When submitting your revision, we need you to address these additional requirements.  1. Please ensure that your manuscript meets PLOS ONE's style requirements, including those for file naming. The PLOS ONE style templates can be found at  https://journals.plos.org/plosone/s/file?id=wjVg/PLOSOne_formatting_sample_main_body.pdf and  https://journals.plos.org/plosone/s/file?id=ba62/PLOSOne_formatting_sample_title_authors_affiliations.pdf. | Revised accordingly throughout the manuscript. | Actioned |
| 2. Thank you for stating the following financial disclosure:  [Unfunded studies].  At this time, please address the following queries:  a) Please clarify the sources of funding (financial or material support) for your study. List the grants or organizations that supported your study, including funding received from your institution.  b) State what role the funders took in the study. If the funders had no role in your study, please state: “The funders had no role in study design, data collection and analysis, decision to publish, or preparation of the manuscript.”  c) If any authors received a salary from any of your funders, please state which authors and which funders.  d) If you did not receive any funding for this study, please state: “The authors received no specific funding for this work.”  Please include your amended statements within your cover letter; we will change the online submission form on your behalf. | As suggested by the editorial office, we clarify that the authors received no specific funding for this work, and the statement of which is included in the cover letter. | Actioned |
| 3. Thank you for stating the following in your Competing Interests section:  [No competing interests].  Please complete your Competing Interests on the online submission form to state any Competing Interests. If you have no competing interests, please state "The authors have declared that no competing interests exist.", as detailed online in our guide for authors at http://journals.plos.org/plosone/s/submit-now  This information should be included in your cover letter; we will change the online submission form on your behalf. | As recommended, we stated in both the previous and current cover letters that the authors have declared that no competing interests exist. | Actioned |
| 4. We note that you have stated that you will provide repository information for your data at acceptance. Should your manuscript be accepted for publication, we will hold it until you provide the relevant accession numbers or DOIs necessary to access your data. If you wish to make changes to your Data Availability statement, please describe these changes in your cover letter and we will update your Data Availability statement to reflect the information you provide. | We decided to provide the raw data as part of the supporting information and included a statement, such as “All relevant data are deposited at [insert a link] and provided within the Supporting Information file,” in the manuscript. | Actioned |
| 5. We note that you have indicated that data from this study are available upon request. PLOS only allows data to be available upon request if there are legal or ethical restrictions on sharing data publicly. For more information on unacceptable data access restrictions, please see http://journals.plos.org/plosone/s/data-availability#loc-unacceptable-data-access-restrictions.  In your revised cover letter, please address the following prompts:  a) If there are ethical or legal restrictions on sharing a de-identified data set, please explain them in detail (e.g., data contain potentially sensitive information, data are owned by a third-party organization, etc.) and who has imposed them (e.g., an ethics committee). Please also provide contact information for a data access committee, ethics committee, or other institutional body to which data requests may be sent.  b) If there are no restrictions, please upload the minimal anonymized data set necessary to replicate your study findings as either Supporting Information files or to a stable, public repository and provide us with the relevant URLs, DOIs, or accession numbers. For a list of acceptable repositories, please see http://journals.plos.org/plosone/s/data-availability#loc-recommended-repositories.  We will update your Data Availability statement on your behalf to reflect the information you provide. | The same as above, we have chosen to include the raw data file as supporting information. | Actioned |
| 6. We note that you have referenced (ie. Bewick et al. [5]) which has currently not yet been accepted for publication. Please remove this from your References and amend this to state in the body of your manuscript: (ie “Bewick et al. [Unpublished]”) as detailed online in our guide for authors  http://journals.plos.org/plosone/s/submission-guidelines#loc-reference-style. | We apologise for any confusion regarding the reference of Bewick et al, as it seems that there may be an error in the citation. We did not cite this reference for our work, as well. However, we removed the two unpublished dissertation works by Ade (1) and Landry (30) and re-numbered the references accordingly in the correct order. |  |
| 7. Please include your tables as part of your main manuscript and remove the individual files. Please note that supplementary tables should be uploaded as separate "supporting information" files.  Additional Editor Comments:  Please, address point-to-point all reviewers' (especially Reviewer 1's and 3's) issues. | We placed the tables at the end of the manuscript.  Thank you so much for bringing these errors to our attention, which we hope to have now corrected all. | Actioned |
|  |  |  |
| Reviewer #1:  First, I would like to commend the authors for doing research in an area where scientists barely have scratched the surface – namely women’s football. However, after a thorough review, it is my opinion that the experiment and statistics in the paper do not meet the technical standards set by the journal criteria. | Thank you for your assessment and comments. On behalf of my co-authors, we sincerely appreciate the time and effort you have dedicated to reviewing and putting into your comments on the initial version of our manuscript. Your reviews have been immensely helpful, and we hope to have addressed the concerns raised by the reviewer and have clarified the points to meet with your expectations. | N/A |
| Under you will find some specific concerns of mine:  Line 100: The description of the participants does not convince me that they are part of an “elite” population. They could, however, be part of an “top-level” population. I would further recommend reading the paper “Defining Training and Performance Caliber: A Participant Classification Framework” by McKay et al (2022). | Thank you for pointing this out. We used the term highly trained football players instead of elite football player according to the classification of McKay et al. (2022). In our specific geographic context, we encounter a challenging grey area to classify as such. This can be attributed to the limited presence of top-tier or world class players, as well as the relatively small number of elite players. Hence, it is possible for highly trained players competing on a national level to occasionally be selected as national level players who play internationally. They are thus provided with a training opportunity required to excel as elite players. This is the background when we refer to elite players, but we made the corrections to ensure clarity on this matter. Thank you for your suggestion. | Actioned |
| Line 102: The sample size calculation does not seem to be correct, and the authors do not specify which statistical test they did the analysis for. Using G*Power software version 3.1.9.7 for a difference between two independent means, with an effect size of 0.3, power of 80%, and alpha at 0.05, I end up with a sample size of 139 in each group! This is very different from the stated minimum sample size of 16. That said, achieving such a sample size (139 in each group) given the population (top-level) is unrealistic. If I could make a suggestion, I would recommend that the authors switch to a Bayesian framework instead. | We thank the reviewer’s observation and have addressed the issue by revising the statement on page 6, accurately reflecting the same size as 176 instead of 16 using G*Power. While we understand the reviewer’s concern regarding the sample size, it is important to note that we carefully considered when determining an appropriate sample size for the study. But ,we also warrant that the findings of the study may be constrained because of the smaller sample size resulting from a limited, lower pool of highly trained female youth footballers. Consequently, we opted to use the standard ANOVA models, as likelihood inferences may yield different conclusions. | Partially actioned |
| Line 110: How the players where randomized is never stated. | Added the information accordingly on page 6, line 110. | Actioned |
| Line 142-145: It is stated that SAQ training was performed with a progressive intensity from 80% to 100%. But you do not state how you measured intensity. Was it RPE or something else?  Testing protocol: The speed testing is both nicely done and described in detail. However, I take issue with the inclusion and execution of the COD and agility tests. First, I looked through the stated references for the reliability of the COD tests (30-32) and the reactive agility test (20). I could not see that these papers set out to determine the reliability of said tests. Furthermore, I would also imagine using a stopwatch would make the timing unreliable even if it is the same rater. Finally, most of the time in the COD and agility tests are spent running instead of changing directions or reacting. It is therefore questionable if these tests are valid and reliable measures of COD or agility. I would also suggest reading some of the literature by Sophia Nimphius on the subject. | Concerning the intensity of the training sessions, heart rate (HR) and rating of perceived exertion (RPE) were consistently monitored. However, we have not documented these records, as they were not within the scope of this study.  We also thank you for your comment regarding the reliability of the COD tests and the reactive agility test here. We appreciate your concern, while we would like to address that the findings of the stated references (30-31) including one that has been removed, proved to provide support for the use of the tests in their respective studies, rather than directly establishing the reliability of the tests. Based on this support, we believe that incorporating the COD tests and reactive agility tests utilised in these prior studies would be appropriate.  Finally, regarding the time of the tests, we understand your concern about using a stopwatch and its potential influence on reliability, even with the same rater. While the use of this mean can introduce some variability, we would also like to acknowledge that we made efforts to minimise such occurrences by ensuring consistent protocols during measurements and testing. We have addressed these inherent limitations in the current version of the study, as well (please see page 19). Thank you once again for bringing up these points, and we regard your feedback is valuable in improving the quality of our study. | Partially actioned |
| Line 243-245: It is first mentioned that pairwise comparisons were not applied because the factors entailed only two groups. This is correct. However, you then go on to say that Bonferroni correction was conducted to determine any significant differences between the two groups or test occasions for each group. This does not make any sense. Also, there is no need to do follow-up independent and paired t-test information in a mixed 2x2 ANOVA – which is actually what you first imply. All the information needed is in the interaction term. | Thank you for pointing this out, and we apologise for any confusion caused by the statement in lines 243-245. We agree with the reviewer’s view that conducing Bonferroni correction for determining differences between the two groups is unnecessary. As a result, we have removed such statements throughout. Additionally, we understand that while the interaction term provides the necessary information, conducting follow-up independent and paired t-tests may still provide supplementary insights, rather than the required, simplifying results obtained from the interaction term.  Reference:  Chang & Yanagisawa (2021). https://doi.org/10.1371/journal.pone.0250326 | Partially actioned |
| Line 254: Perhaps nitpicky, but you should state whether you used the pre-test and post-scores, or the change scores, to determine normality. The former is incorrect, while the latter is correct. | We performed Shapiro-Wilk and Levene’s tests to determine normality and homogeneity of variances using the residual terms of change scores and added such information in the manuscript. | Actioned |
| Line 255: I suspect you did an independent t-test at baseline to check that the randomization procedure worked correctly. However, one should actually never do this. The more tests you do the larger probability that one of the tests turns is significant purely by chance. | We acknowledge that significance testing of baseline differences in randomized controlled trials is not performed in many fields, while others require these tests, although they may be superfluous. While reaching a definite resolution on this matter has been challenging to us, it is worth discussing why we insist on conducting them. Through randomisation, we ensured the balancing of participant characteristics between the groups. We would like to investigate the effects of SAQ training on female youth players instead of testing the effectiveness of randomisation procedures. Given the certain factors specific to our research context, we aim to address potential influences of baseline differences on the study outcomes. We have therefore chosen to include these tests to offer meaningful information to our readers. | N/A |
| Results section: As mentioned previously, the independent and paired t-test are redundant and should be removed. Instead, I would suggest that you report the mean change ± 95% confidence intervals for both groups, and the mean difference ± 95% confidence intervals between the groups. | Thank you for your comment. In response to the reviewer’s suggestion, we have included the information in Table 2. | Actioned |
|  |  |  |
| Reviewer #2:  The aim of the present study was to investigate the effect of an 8-week speed, agility, and quickness (SAQ) training on linear sprint speed, change of-direction speed, and reactive agility in elite U-20 female football players. The study is well written, easy to read, and provides new insights into the potentiality of SAQ training for improving various physical features of soccer players. Introduction is clear and provides an appropriate description of background and rationale. Methods are well described. Results section can be improved in terms of its readability (although I understand that describing results of two way anova may not be easy). Discussion provides a depth analysis of results, in light of the literature on SAQ methodology. I would congratulate the Authors for their work. I have no particular comments, apart from minor ones that I hope may be useful to improve the overall quality of the manuscript. | We appreciate the reviewer’s comment on providing positive, encouraging comments and suggestions to improve the readability of the results section. In response, we have carefully revised the content of the results section to set up readers to better understand the findings of the study. To ensure clarity, we have also taken the time to thoroughly proofread and rephrase the manuscript where necessary. | Actioned |
| Line 47-49. The contrary? Maximum speed 10-30 m; acceleration 0 to 5 m. | Thank you bringing this oversight to our attention. We corrected the statement by adding “acceleration” between maximum and speed. | Actioned |
| Line 152. Were the players familiarized with the testing protocols? | Before commencing the 8-week training period, we ensured that all players were familiar with the testing and training procedures, as stated in our earlier version of the manuscript. | N/A |
| Table 3 is appropriate. However, I would suggest to consider to substitute the table with graphs, that would allow an immediate visual impression of the behaviour of the variables tested for the two groups. I understand that including graphs for all the variables may be impractical, but graphs for some variables (the most important ones) may be useful. Moreover, please insert units of measurements for the variables in the table (for examples speed). | Thank you for your comment. We agree with your point that graphical representations can provide with a more insightful understanding of the findings of study. Hence, we have taken your suggestion into consideration and added the bar graphs of changes in performance and its interaction term to further enhance the visual representation of the data. | Actioned |
|  |  |  |
| Reviewer #3: The aim of the study was investigating the effects of a relatively long training program based on SAQ on physical performance in elite female football players.  The manuscript cover an interesting topic, providing additional information on the usefulness of SAQ for improving performance in a female sample of soccer players.  However, I would suggest the authors to improve the readability of the text by better proofreading the English language. Moreover, they are requested to better improve the methodological aspects in order to support what have been done. Lastly, they are also recommended to better explore the literature reporting a certain specificity between COD performance and acceleration. IMO, the completeness of the discussion section would benefit from such exploration. | We greatly appreciate the reviewer’s suggestion to improve the readability of the manuscript. In response to Reviewer 2’s similar feedback, we have made amendments to the manuscript to improve the readability and accessibility throughout. We believe these changes on the methods section make it easier for readers to understand and more engaging. Finally, we also added some sentences to the discussion section to clarify interpretation of the results. | Actioned |
| Here below some specific comment  Specific comments  Abstract  line 17: suggested rewording -->"the effects of an 8-week speed, agility, and quickness (SAQ) training on..." | We revised the statement accordingly. | Actioned |
| line 21: "The players were tested for:" | We revised the statement accordingly. | Actioned |
| Intro  lines 37-38: "...actions (e.g., passing kicking, trapping, dribbling, tackling) and without ball (...)" | We revised the statement accordingly. | Actioned |
| line 49: (0 to 5 m) | We revised the statement accordingly. | Actioned |
| line 52: reactive is redundant. The term agility already embodies cognitive skills in which reaction plays a role. | We revised the statement accordingly. | Actioned |
| lines 76-78: please rephrase for clarity | We revised the statement accordingly. | Actioned |
| line 86-87: please rephrase for clarity. Perhaps also replace "researchers know little" to "little is known..." | We revised the statement accordingly. | Actioned |
| M&M  lines 106-109: state clearly all inclusion and exclusion criteria | We have stated the inclusion and exclusion criteria in the previous draft of this manuscript. | N/A |
| line 133: remove "were" | We revised the text accordingly. | Actioned |
| line 134: I would move this sentence from here. Outside the context. | Thank you for your suggestion. However, we revised the sentence to make it clear, instead. | Partially actioned |
| line 135: "normal dietary"? Please be specific. Perhaps the following ref may serve to provide more detail abut that statement.  doi: 10.1080/15438627.2020.1809410 | We have included the information as suggested by the reviewer. | Actioned |
| line 145: how did the authors control the intensity? | Thank you for your question. Reviewer 1 was also concerned how we measured the intensity. In response, we provided an explanation as follows:  “Concerning the intensity of the training sessions, heart rate (HR) and rating of perceived exertion (RPE) were consistently monitored. However, we have not documented these records, as they were not within the scope of this study.” | Partially actioned |
| line 171: please add reference for such a choice | We have added a reference on page 9. | Actioned |
| Results  I would suggest the authors to first stating the outcomes of the interactions. | We revised the results section based on the suggestions provided by the reviewer. | Actioned |
| Discussion  In the first part of this section, IMO, It is important to clarify whether the study hypothesis was verified. | We appreciate the reviewer pointing out this. In response, we have included such a statement explaining that our hypothesis was supported by the test. We believe this addition provides clarity and strengthens the overall findings of our study. | Actioned |
| lines 364-366:  this should be moved at the beginning of the introduction section, after the main findings are stated. | Thank you for your suggestion. However, we would like to clarify that, in our attempt to discuss the findings of reactive agility test, we have incorporated relevant findings from previous studies. By doing so, we aimed to provide a well-rounded analysis of the area. Thus, we believe this statement serves as a valid marker for highlighting the significance of our discussion and showing its alignment with existing research. | N/A |
| line 381: What about the studies suggesting that acceleration is independent of COD performance? Please consider the works of Nimphius and other authors, for example.  doi: 10.1519/JSC.0000000000001421  DOI: 10.1519/SSC.0000000000000309  doi: 10.7717/peerj.9486 | We appreciate the reviewer’s comment and the references provided. Taking into consideration the reviewer’s earlier point, we have added such a discussion to elaborate on the different techniques required for players to develop and assess their sprinting and COD speed (on page 18). We believe this addition in the discussion section has provided further insights into the various skills and strategies involved in improving athletic abilities.  Again, we thank you for your precious time in reviewing our manuscript and providing valuable comments. Your feedback has been instrumental in improving the current version of the manuscript. | Actioned |
