# Supplementary material for: SAQ training on sprint, change-of-direction speed, and agility in U-20 female football players
Source: PLoS One. 2024 Mar 13;19(3):e0299204. doi: 10.1371/journal.pone.0299204 (PMC10936847; doi:10.1371/journal.pone.0299204)
Supplement: Attachment — Submitted filename: Plos_SAQ_responses_to_reviewers_R2.docx [file pone.0299204.s003.docx]

PONE-D-22-09664 “SAQ training on sprint, change-of-direction speed, and agility in U-20 female football players”

Point-by-point responses to the reviewers’ comments

Overall comments: We express our gratitude to the editors for allowing us to submit our revised draft of the manuscript and to the reviewers for their valuable and constructive comments. Your suggestions have greatly contributed to the enhancement of the current manuscript. We carefully analysed the feedback to address the concerns raised by the reviewers and worked to strengthen those aspects. The right column below provides a point-by-point description of each change we made (next to the actioned one), and we have marked the edits in the manuscript using the track changes function in Word.

| Reviewers’ comments | Responses | Actioned |
| --- | --- | --- |
| Journal Requirements:  Please review your reference list to ensure that it is complete and correct. If you have cited papers that have been retracted, please include the rationale for doing so in the manuscript text, or remove these references and replace them with relevant current references. Any changes to the reference list should be mentioned in the rebuttal letter that accompanies your revised manuscript. If you need to cite a retracted article, indicate the article’s retracted status in the References list and also include a citation and full reference for the retraction notice | Thank you for the suggestion. We ensured the reference list thoroughtly and updated accordingly. | Actioned |
| Additional Editor Comments:  Please, one further effort. Address point-to-point Reviewer 1 and 3' issues. | We addressed the issues raised by Reviewer 1 and 3 and made the necessary amendments in the revised manuscript. | Actioned |
|  |  |  |
| Reviewer #1:  I think the authors have done a superb job in updating the manuscript, which is almost ready for publication.  I only have a few comments: | Thank you for your positive feedback on the updated manuscript. On behalf of my co-authors, we express our deep gratitude to you and the other reviewers for your valuable time and effort in reviewing and providing us with feedback. We have strived to address the concerns raised by the reviewer 1 and clarify the points to meet your expectations. | N/A |
| 1) I recommend changing the interaction plot to a pre-post dot plot so that we could see each data point. For an example see figure 4.1 in <https://mladenjovanovic.github.io/bmbstats-book/causal-inference.html> | Thank you for your suggestion to change the interaction plot to a pre-post dot plot. While we appreciate your idea, we believe that the current interaction plot better captures the overall trend and pattern of the data. We think that a pre-post dot plot may provide more detailed visible individual data points, the dot plot might result in a cluttered and less interpretable. Hence, we believe the current interaction plot allows for a clearer visualization of the relationship between variables and a more concise and informative representation of the data compared to a pre-post dot plot, helping us identify and interpret the overall significant interaction effects. During this process, we have identified critical errors in the interaction plots for arrowhead dribble (left and right), which are now corrected properly. We appreciate your attention to this matter, and if there are specific concerns or limitations you have identified with the current plot, we would be happy to discuss them further and explore potential solutions. However, at this time, we believe that sticking with the interaction plot provides the clearest presentation of the data for our purposes. Thank you! | N/A |
| 2) Could you write a sentence or two in the manuscript of how intensity was evaluated? You noted in a previous comment that RPE and Heart Rate were measured, but out of the scope of the paper and thus not reported, which is fine. However, some explanation of how intensity was evaluated and communicated to the athletes should appear in the text. | We also thank you for your comment regarding the absence of information concerning the intensity of the training sessions. Based on your suggestion, we have added this information discussing the monitoring of heart rate and rating of perceived exertion during the training sessions on page 8, line 152-154.We have also included a discussion that acknowledges its limitations of the study on page 19. We highlighted how this limitation may impact our findings and the potential implications for future studies. We appreciate your attention to detail and thank you again for bringing this to our attention. | Actioned |
| 3) I'm sorry, but I fail to see how the references (30-31) underscore the validity and reliability of the tests that have been used:  Wing, C. E., Turner, A. N., & Bishop, C. J. (2020). Importance of strength and power on key performance indicators in elite youth soccer. The Journal of Strength & Conditioning Research, 34(7), 2006-2014.  Joo, C. H. (2016). The effects of short-term detraining on exercise performance in soccer players. Journal of exercise rehabilitation, 12(1), 54.  As far as I know, the sprint tests along with the dribbling/slalom tests are both valid and reliable (see: Altmann, S., Ringhof, S., Neumann, R., Woll, A., & Rumpf, M. C. (2019). Validity and reliability of speed tests used in soccer: A systematic review. PloS one, 14(8), e0220982.). Arrowhead is reliable but it's validity has not been tested. SEMO has neither been tested for validity and reliability, but I can agree that it is widely used. Please address this in the text. | Thank you for bringing up this point and providing the specific information with clarification on the validity and reliability of the tests. We understand your concern regarding the lack of validity testing for the arrowhead test and the absence of both validity and reliability testing for the SEMO test. Hence, we included texts on page 20, highlighting the limited available evidence regarding the validity and reliability of the arrowhead test. We also mentioned that the SEMO test is widely used but has not yet been tested for validity and reliability. By explicitly stating these limitations, we aim to provide a balanced and accurate representation of the current knowledge on these tests.  We appreciate again for your suggestion to address this issue in the manuscript. Your contribution is instrumental in ensuring that our work reflects the most accurate information. | Actioned |
| 4) This might be semantics on my part, but I don't like the arrowhead and SEMO to be defined as COD or agility tests. I think they are more tests of maneuverability, as they can't really isolate the COD or agility component. I would be happy if this is addressed in the text, but feel free to leave the definitions as is. | Thank you for your input regarding the definitions of the arrowhead and SEMO to be defined as COD or agility tests. We understand your perspective that these tests may be more accurately described as tests of manoeuvrability rather than specifically isolating the COD or agility component. We appreciate your flexibility in leaving the definitions as they are, despite considering the potential semantics.  To address this concern and provide further clarity in the text, we have revised section on page 4 and added some additional context from previous studies. This addition highlights the multifaceted nature of these tests and acknowledge that while they may not solely isolate the COD or agility component, they do evaluate an individual’s maneuvring ability through the SAQ training. Again, we appreciate your feedback to consider nuances in the definitions and regard your points are valuable in improving the quality of the study. | Partially actioned |
|  |  |  |
| Reviewer #2:  No response |  | N/A |
|  |  |  |
| Reviewer #3:  The authors have put a lot of effort in addressing the Reviewer's point. The manuscript has considerably improved. However, I feel that some important recent references are still missing. Please see the following:  doi: 10.1371/journal.pone.0277683  doi: 10.3390/ijerph18041962  These would help the authors to reinforce both the background and rationale of the study. | Thank you for acknowledging our efforts in addressing the Reviewers’ points in the previous and current version of the manuscript and stating that the manuscript has significantly improved. Your concern regarding the absence of recent important references is duly noted, and we added them on page 4 to strengthen our study background. By including these additional sources, we aim to enhance the overall quality and relevance of our work.  We sincerely appreciate your valuable point, which has helped us improve the work. If you have any further suggestions or concerns, we would be more than happy to address them accordingly. Thank you! | Actioned |
